# The Effect of Conditioned Medium from Angiopoietin-1 Gene-Modified Mesenchymal Stem Cells on Wound Healing in a Diabetic Mouse Model

**DOI:** 10.3390/bioengineering11121244

**Published:** 2024-12-09

**Authors:** Qiong Deng, Shenzhen Pan, Fangzhou Du, Hongfei Sang, Zhixin Cai, Xiaoyu Xu, Qian Wei, Shuang Yu, Jingzhong Zhang, Chenglong Li

**Affiliations:** 1 Department of Vascular Surgery, The Second Affiliated Hospital of Soochow University, Suzhou 215000, China; dengqiong1028@163.com (Q.D.); pansz1998@163.com (S.P.); sanghongfei@163.com (H.S.); czhxinsx@163.com (Z.C.); 2 School of Biomedical Engineering (Suzhou), Division of Life Sciences and Medicine, University of Science and Technology of China, Hefei 230026, China; du_fangzhou@163.com (F.D.); qianwei@mail.ustc.edu.cn (Q.W.); yush@sibet.ac.cn (S.Y.); 3 Suzhou Institute of Biomedical Engineering and Technology, Chinese Academy of Sciences, Suzhou 215163, China; 4 Department of Radiology, Xiangyang Central Hospital, Affiliated Hospital of Hubei University of Arts and Science, Xiangyang 441000, China; xuxiaoyu19941015@163.com

**Keywords:** mesenchymal stem cell, conditioned medium, Angiopoietin 1, diabetic wound healing, angiogenesis

## Abstract

Introduction: Mesenchymal stem cells (MSCs) have been introduced as a promising treatment for diabetic wounds. The effects of stem cell therapy are thought to be caused by bioactive molecules secreted by stem cells. Stem cell-based gene therapies can target bioactive molecules. Therefore, treatment using conditioned medium (CM) derived from genetically engineered stem cells has been proposed as an alternative option for diabetic ulcer care. Methods: MSCs derived from human umbilical cords were obtained and engineered to overexpress the angiogenin-1 gene (MSCs^Ang1^) through plasmid transfection. This study extracted conditioned medium from MSCs (MSC-CM) or MSCs^Ang1^(MSC^Ang1^-CM) for wound treatment applications. Via in vitro experiments, the proangiogenic effects of MSC^Ang1^-CM were assessed via the migration and tube formation of human umbilical vein endothelial cells (HUVECs). Furthermore, the efficacy of MSC^Ang1^-CM in promoting wound healing, re-epithelialization, hair follicle, and angiogenesis was evaluated via a diabetic mouse skin defect model. Results: In vitro assays demonstrated that MSC^Ang1^-CM significantly enhanced HUVECs’ functions, including migration and tube formation. In vivo assays revealed that MSC^Ang1^-CM exhibited notable advancements in healing speed, re-epithelialization, hair follicle, and angiogenesis. Conclusion: These results indicate that MSC^Ang1^-CM can promote wound healing in diabetic mice and make the vascular structure in regenerated tissues more stable without inducing tissue fibrosis, providing a new therapeutic strategy for treating diabetic skin wounds. This provides a valuable theoretical basis for further research on regenerative medicine and cell therapy.

## 1. Introduction

The global incidence of type 2 diabetes is on a steady rise and currently affects more than 500 million patients worldwide [1]. Approximately 20% of these patients suffer from non-healing wounds on the lower limbs or feet, with severe cases often leading to necrosis or even amputation. It not only brings immense suffering for the patients but also causes huge economic costs [2]. Currently, the primary treatments for diabetic wounds include control of blood glucose levels, dietary modification, and local wound care. However, these treatments have limited effects and diabetic foot ulcer remains a challenge in current clinical treatment.

Studies have shown that diabetes results in reduced angiogenesis within wounds, leading to delayed healing in diabetic patients [3,4]. A reduction in the expression of angiogenesis-regulating genes, such as vascular endothelial growth factor (VEGF) and Angiopoietin-1 (Ang1), is a significant factor behind reduced angiogenesis [5]. Consequently, strategies to stimulate angiogenesis have become a focal point of research aimed at accelerating wound healing in DFU [6]. Ang1, a 70 kDa glycoprotein in the angiopoietin family, is synthesized by perivascular support cells, including pericytes, vascular smooth muscle cells, and tumor cells. Acting as a natural agonist for the TIE-2 receptor, Ang1 specifically binds to and promotes the phosphorylation of the TIE-2 receptor on endothelial cells through paracrine action, facilitating the maturation of new blood vessels [7,8]. Ang1 is crucial for improving impaired angiogenesis and wound healing in diabetes [9], playing a vital role in vascular regeneration [10]. Elevating Ang1 levels at the wound area has been identified as an effective measure to mitigate reduced angiogenesis in diabetes [11]. However, the direct application of cytokines, despite being effective in treating diabetic ulcers, is still facing challenges such as uncontrolled dosage due to cytokine inactivation, degradation, and huge economic burden [12]. Therefore, it is necessary to identify appropriate carriers to circumvent the limitations of directly applying Ang1 to diabetic patients.

MSCs possess multidirectional differentiation potential and self-renewal capabilities, presenting vast potential in diabetic wound treatment [13]. Recent studies delve deeper into using exogenous stem cell transplantation to enhance wound healing [14,15,16]. MSCs mainly facilitate tissue regeneration in wounds through paracrine action, secreting a cocktail of bioactive factors known as the MSC secretome or CM [17]. Factors produced by MSC paracrine action, such as Ang1 and VEGF, play crucial roles in the angiogenesis process of diabetic wounds. MSC-CM, being easy to extract, cost-effective, clinically practical, rich in factor content, and low in immunogenicity, has emerged as a novel and promising treatment method, representing a forward-looking and optimal therapy modality for MSCs [18]. However, MSCs alone do not produce sufficient Ang1 and exogenous Ang1 is prone to degradation and inactivation in the local wound environment.

MSCs modified with the Ang1 gene may help overcome these problems. Ang1-modified bone marrow mesenchymal stem cells not only continuously release Ang1 in situ but also provide cells and cytokines, creating an appropriate environment to accelerate wound healing in rat models [19]. By designing an Ang1 plasmid and infecting MSCs with a lentivirus, we obtained MSCs^Ang1^ and collected MSC^Ang1^-CM for local treatment of diabetic mice wounds. Thus, this study designed a novel acellular therapy, using the CM from MSCs^Ang1^ to treat wounds in diabetic mice. This study aims to elucidate the role of the devised engineered MSC^Ang1^-CM on wound healing in diabetic mice.

## 2. Materials and Methods

### 2.1. Preparation of the Primary MSCs and Culture

All research involving human samples was conducted in strict compliance with the ethical guidelines for human embryonic stem cell research and the Declaration of Helsinki. Umbilical cord samples were collected with informed consent signed by donors and ethical approval obtained from the First Affiliated Hospital of Soochow University (Approval No. 2019-136). MSCs were isolated and cultured following previously established protocols [20]. Briefly, small fragments of Wharton’s jelly were placed in Dulbecco’s Modified Eagle Medium/Nutrient Mixture F-12 (DMEM/F12; Gibco, Grand Island, NY, USA; C11330500BT) supplemented with 10% fetal bovine serum (FBS; Gibco, Grand Island, NY, USA; 10099-141) and 1% penicillin-streptomycin solution (P/S; Solarbio, Beijing, China; P1400).

### 2.2. Lentivirus Production and Preparation of GFP-Labeled MSCs That Overexpressed Ang1

The GFP-labeled Ang1 lentivirus was produced in human embryonic kidney 293T cells co-transfected with the pLVX CMV-Ang1-EGFP lentiviral vector (Miaoling, Wuhan, China) and three packaging plasmids, as previously described. Viral particles were collected and concentrated by ultracentrifugation at 50,000× *g* for 3 h at 4 °C using a Thermo LYNX-6000 (Thermo, Tokyo, Japan) centrifuge. The resulting pellet was gently resuspended in ice-cold PBS, aliquoted, and stored at −80 °C for later use. For transduction, MSCs at passages 0 to 2 (P0–P2) with approximately 80% con-fluence were incubated with the Ang1 lentivirus in D/F medium containing 8 μg/mL polybrene (Yeasen, Shanghai, China; 40804ES) for 6 h. Following this, the medium was replaced with D/F supplemented with 10% FBS and 1% P/S for an additional 24 h. After 48 h, the efficiency of Ang1 overexpression was assessed using GFP immunofluorescence staining. MSCs with GFP-labeling efficiency exceeding 70% were selected for subsequent transplantation experiments.

### 2.3. Preparation of the Conditioned Medium

To prepare the CM, MSCs and MSCs^Ang1^ (P3–P6) were first washed thoroughly with PBS (Gibco; C10010500BT) and then incubated in serum-free D/F medium. After 48 h, the supernatants (MSC-CM and MSC^Ang1^-CM) were collected and subjected to centrifugation at 2000× *g* for 10 min at 4 °C to eliminate cell debris. The clarified supernatants were subsequently filtered through a 0.22 μm membrane filter (Merck Millipore, Billerica, MA, USA; SLGP033RB) and stored at −80 °C for later use. CM was defined as the final processed supernatant, while D/F medium incubated under the same conditions without MSCs was used as the Control.

### 2.4. Preparation of the Diabetic Mice Wound Model

All animal experiments adhered to the ARRIVE (Animal Research: Reporting of In Vivo Experiments) guidelines and were approved by the Biological Research Ethics Committee of the Chinese Academy of Sciences (Approval No. 2021-B13). The mice were housed in a specific pathogen-free environment under a 12 h light/dark cycle. Adult male C57BL/6J mice (7–8 weeks old) obtained from Cavens Lab Animals Co., Ltd. (Changzhou, China) were maintained on a high-fat diet (60 kcal% fat, FBSH, Wuxi, China) for one week before undergoing a streptozotocin (STZ; 50 mg/kg/day; AbMole, Shanghai, China; M2082) intraperitoneal injection for 5 consecutive days. Blood glucose levels were measured every fifth day post-STZ injection using a glucometer (Accu-Chek Performa, Roche Diagnostics, Shanghai, China) via tail vein sampling. Mice with fasting blood glucose levels of ≥16.7 mM for two weeks were classified as diabetic and selected for the subsequent wound-healing experiments. At 12–13 weeks of age, full-thickness skin wounds were created after depilation under anesthesia induced by 3% isoflurane (RWD, Shenzhen, China; R510-22). Using a skin biopsy punch, two circular skin defects, each 8 mm in diameter and spaced at least 1 cm apart, were created on the dorsal surface. The wounds were covered with an antimicrobial dressing (Drape Antimicrob, Saint Paul, MN, USA; REF6640) to ensure sterility and minimize skin contraction.

### 2.5. Animal Treatment and Wound Area Evaluation

Eighteen diabetic mice were randomly assigned to three treatment groups, receiving either PBS (20 μL), MSC-CM (20 μL), or MSC^Ang1^-CM (20 μL). Each wound was treated with the corresponding solution immediately after wound creation (D0) and subsequently every three days to maintain therapeutic efficacy. Wound sizes were documented on days 0, 3, 7, and 14 post-treatment using a digital camera positioned perpendicularly to the wound. The captured images were analyzed using FIJI [21] with standardized calibration to calculate wound area and assess healing progress.

### 2.6. Immunohistochemistry

Mice were euthanized via CO_2_ inhalation and wound specimens (Φ = 8 mm), including the wound bed and adjacent regenerated skin tissue, were excised and bisected along the midline. The left half was allocated for total RNA or protein extraction, while the right half was fixed in 4% paraformaldehyde (Sigma, St. Louis, MO, USA; 158127), embedded in paraffin, and sectioned at a thickness of 6 μm. These sections underwent hematoxylin and eosin (H&E) staining (Solarbio, Beijing, China; G1120) or immunofluorescence staining. For immunofluorescence, the sections were incubated overnight at 4 °C with primary antibodies, including anti-CD34 (Abcam, Cambridge, MA, USA; ab8158), anti-K14 (Abcam, Cambridge, MA, USA; ab181595), anti-K17 (Santa Cruz, CA, USA; sc-393091), and anti-α-SMA (ab5694). Immunoreactivity was detected using appropriate Alexa Fluor-conjugated secondary antibodies, and imaging was performed using a confocal microscope (Nikon, Tokyo, Japan; A1R HD25). Structural evaluations were conducted in randomly selected fields within the transitional zones of the wound area.

### 2.7. Scratch Assay

To evaluate the migration of HUVECs, a scratch test was performed. A 200 μL pipette tip was used to create a scratch in the cell monolayer cultured in 6-well plates. The cells were then incubated in D/F medium supplemented with either 50% CM or Control medium. At various time points, images of the scratches were captured using a microscope. The extent of cell migration was quantified by analyzing the uncovered area with Image J software (NIH, Bethesda, MD, USA). The migration rate was determined using the following formula: migration rate = [(initial area − uncovered area)/initial area] × 100%.

### 2.8. Transwell Migration Assay

A transwell migration assay was performed to study the endothelial migration using 24-well transwell plates (Corning, NY, USA; 3422) containing 8 μm pore size polycarbonate filters. Briefly, the cultured HUVECs were trypsinized and seeded at the density of 1 × 10^5^ cells suspended in 100 μL serum-free medium in the upper well. D/F medium containing 50% CM or Control was added to the lower chamber of the transwell system. After 24 h of incubation, migrated cells were fixed with 4% paraformaldehyde and stained with 0.1% crystal violet (Solabio, Beijing, China; G1063) for 20 min at room temperature. Images were acquired using a microscope and counted. Cell migration is expressed as % of Control.

### 2.9. Tube Formation Assay

The wells of 96-well plates were covered with 60 μL Matrigel (Corning, NY, USA; 354277) after 30 min of incubation at 37 °C of 2 × 10^4^ cells suspended in D/F medium containing 50% CM or Control and added to the plate. After 4 h of incubation at 37 °C, the capillary-like structures were visualized with a microscope and photographed to record, and the total tube length was quantified by FIJI [21].

### 2.10. Quantitative PCR (q-PCR)

Total RNA from wound skin samples (left half) or cells treated with different treatments was extracted with the Trizol (Ambion, Austin, TX, USA; 15596026) method and quantified with a NanoDrop2000 (Thermo, Waltham, MA, USA). q-PCR was performed using TB Green Premix Ex Taq (TAKARA, Tokyo, Japan; RR042B) and appropriate primers (Table S1) on a Bio-Rad CFX96 PCR system. The housekeeping gene GAPDH was used as an internal reference.

### 2.11. Western Blot

The left half of the wound tissue or cultured cells was lysed in cell lysis buffer (Beyotime, Shanghai, China; P0013). Protein concentrations were measured using a BCA Protein Assay Kit (Thermo, Shanghai, China; PA115-01). Equal amounts of protein were resolved on 10–15% SDS-PAGE gels and transferred onto polyvinylidene fluoride (PVDF) membranes. The membranes were blocked and incubated overnight at 4 °C with primary antibodies targeting Ang1 (Abcam, Cambridge, MA, USA; ab183701) and CD34 (Abcam, Cambridge, MA, USA; ab8158), followed by incubation with Alexa Fluor-conjugated secondary antibodies. Protein bands were detected using an enhanced chemiluminescence kit (GE Life Sciences) and quantified with FIJI [21] after correcting for local background noise.

### 2.12. Statistical Analysis

All numerical data are expressed as mean ± SD. Statistical analyses were performed using unpaired t-tests or analysis of variance (ANOVA) followed by appropriate post hoc tests, utilizing GraphPad Prism software (version 8.0, GraphPad Software, La Jolla, CA, USA). A *p*-value of less than 0.05 was considered statistically significant.

## 3. Results

### 3.1. Construction of Engineered MSCs^Ang1^ and Preparation of Conditioned Medium

**Figure 1:**
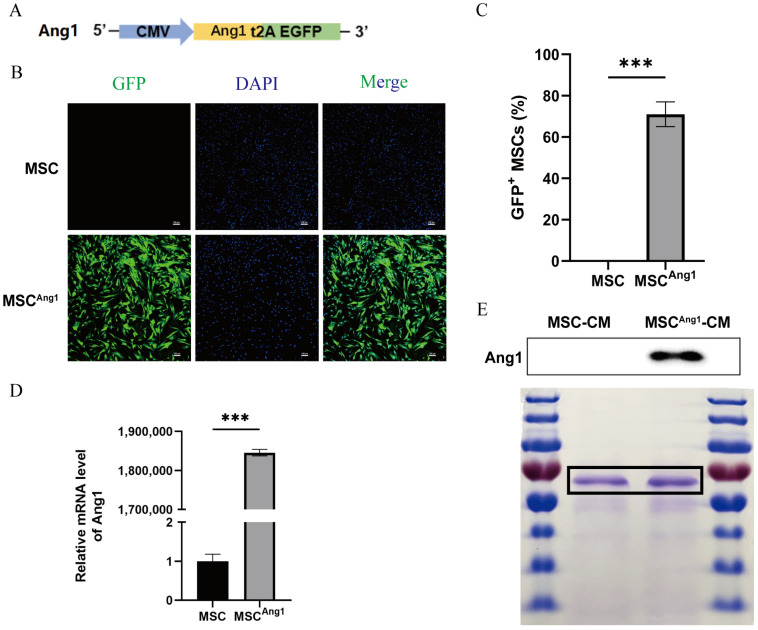
Construction of MSCs^Ang1^ and preparation and characterization of CM. (**A**) Schematic representation of the lentiviral plasmid carrying Ang1 and GFP linked via a T2A peptide. (**B**) Immunofluorescence staining of GFP in MSCs^Ang1^. (**C**) Quantitative analysis of GFP fluorescence intensity in MSCs^Ang1^ showed a high GFP-positive expression rate. (**D**) qPCR analysis revealed that the mRNA expression level of Ang1 in MSCs^Ang1^ was significantly elevated compared to MSCs. (**E**) Ang1 protein levels in MSC-CM and MSC^Ang1^-CM were assessed, demonstrating a significant increase in Ang1 expression in MSC^Ang1^-CM relative to MSC-CM. Total protein content in MSC-CM and MSC^Ang1^-CM was quantified using Coomassie Brilliant Blue staining. Scale bar: 100 μm. All data are presented as mean ± SD. *** *p* < 0.001.

A lentiviral plasmid designed to overexpress Ang1, linked to GFP via a T2A peptide, was successfully constructed (Figure 1A). MSCs engineered to overexpress Ang1 (MSCs^Ang1^) were generated through lentiviral infection. Immunofluorescence staining for GFP revealed that approximately 73% of the MSCs^Ang1^ were GFP-positive (Figure 1B,C), indicating successful viral transduction. The expression level of Ang1 mRNA was evaluated using qPCR, which demonstrated a significant upregulation of Ang1 in MSCs^Ang1^ compared to MSCs (Figure 1D). These findings confirmed the successful generation of the engineered MSCs^Ang1^. Subsequently, we assessed Ang1 protein levels in the CM via western blotting and quantified the total protein content in the CM using Coomassie Brilliant Blue staining (Figure 1E). The results showed that the Ang1 protein expression in MSC^Ang1^-CM was significantly enriched relative to the total protein content compared to MSC-CM, confirming the successful preparation of engineered MSC^Ang1^-CM.

### 3.2. Engineered MSC^Ang1^-CM Stimulates HUVECs Migration and Tube Formation In Vitro

**Figure 2:**
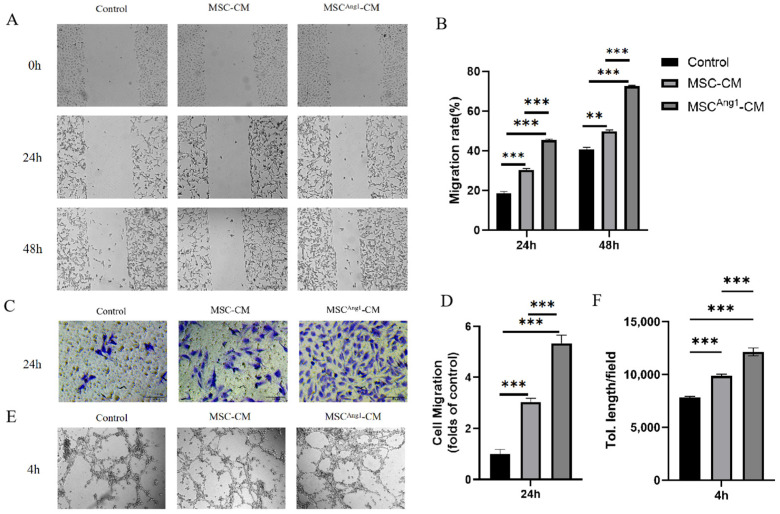
Effects of different treatments on HUVECs’ migration and tube formation. (**A**) Images of HUVECs’ migration changes after 24 h and 48 h of different treatments. (**B**) Quantitative statistical analysis of cell migration rate showed that compared to Control and MSC-CM, MSC^Ang1^-CM significantly promoted HUVECs’ migration. (**C**) Number of HUVEC migrations in the transwell chamber after 24 h of different treatments. (**D**) Quantitative statistical analysis of the number of cell migrations shows that MSC^Ang1^-CM significantly promoted the number of HUVEC migrations compared to Control and MSC-CM. (**E**) Tubulogenic images of HUVECs after treatment with Control, MSC-CM, and MSC^Ang1^-CM for 24 h. (**F**) Quantitative statistical analysis of tubulogenesis experiments showed that compared to MSC-CM and Control, the total length of tubes in the MSC^Ang1^-CM group was significantly increased, with statistically significant differences observed among the three groups. Control: Replacement of 50% of the original medium of HUVECs with fresh D/F medium. Scale bar: 100 μm. All data are presented as mean ± SD. ** *p* < 0.01, *** *p* < 0.001.

To investigate the possible role of MSC^Ang1^-CM in endothelial angiogenesis, HUVECs were cultured and treated with CM or the Control (Figure 2A). We detected the migration of HUVECs in vitro. The data demonstrate that HUVECs’ migration was significantly accelerated in MSC^Ang1^-CM compared to the Control and MSC-CM at both 24 h and 48 h (Figure 2B). We also examined migration activity by using a transwell migration assay (Figure 2C). Similar results were observed in the transwell migration assay (Figure 2D). Next, we tested the role of MSC^Ang1^-CM in the formation of 2D capillary tubes in HUVECs, another property related to angiogenesis. By using the 2D Matrigel assay, it was shown that MSC^Ang1^-CM induced the formation of a well-organized, capillary-like network (Figure 2E). The data suggested that the tube length was more enhanced in the MSC^Ang1^-CM condition compared to the Control and MSC-CM (Figure 2F). Together, these data revealed that MSC^Ang1^-CM can induce angiogenesis in vitro by enhancing the migration and tube formation ability of HUVECs.

### 3.3. Engineered MSC^Ang1^-CM Accelerates Wound Healing in Diabetic Mice

**Figure 3:**
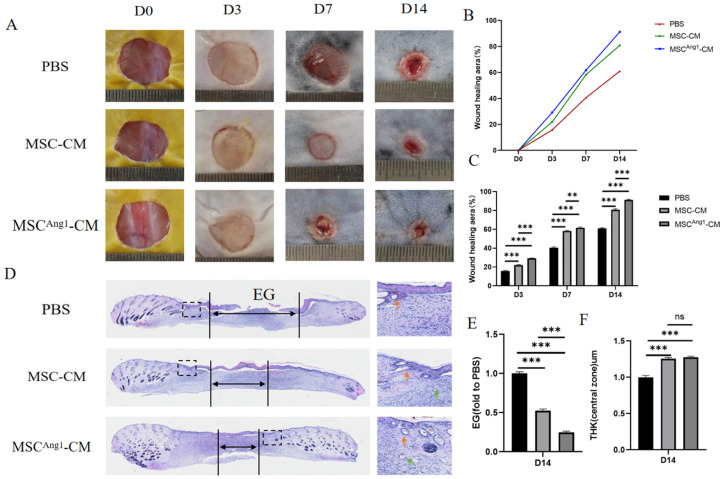
Effect of MSC^Ang1^-CM on wound healing in diabetic mice. (**A**) Dynamic changes and (**B**) healing rates of diabetic mice wounds under different treatments. (**C**) Quantitative statistical analysis of dynamic changes in wound healing showed that compared with PBS and MSC-CM groups, the MSC^Ang1^-CM treatment group significantly accelerated the healing of skin wounds in diabetic mice. (**D**) H&E staining of diabetic mice wound tissues after 14 days of different treatments, orange arrows indicate hair follicles, and green arrows indicate blood vessels. (**E**) Quantitative statistical analysis of the Epithelial Gap (EG) in diabetic mice wounds after 14 days of treatments with PBS, MSC-CM, and MSC^Ang1^-CM showed that compared with PBS and MSC-CM EGs, the EG in the MSC^Ang1^-CM treatment group was significantly reduced and the re-epithelialization of skin wounds was significantly accelerated. (**F**) Quantitative statistical analysis of wound thickness in diabetic mice on day 14 showed that the skin thickness in the MSC-CM and MSC^Ang1^-CM groups was increased compared to the PBS group, while there was no significant difference between the MSC-CM and MSC^Ang1^-CM groups. All data are presented as mean ± SD. ns *p* > 0.05, ** *p* < 0.01, *** *p* < 0.001.

Next, we evaluated the effects of MSC^Ang1^-CM on promoting wound healing; diabetic mice wounds were randomly divided into groups and received treatments of PBS, MSC-CM, and MSC^Ang1^-CM every three days. Compared to the PBS and MSC-CM mice, MSC^Ang1^-CM diabetic mice showed significantly smaller wound areas at 3 days, 7 days, and 14 days after different treatments (Figure 3A–C). The area not covered by the epidermis, defined as the Epithelial Gap (EG), was markedly smaller in the MSC^Ang1^-CM group compared to that in PBS and MSC-CM groups after 14 days, showing statistical significance (Figure 3D,E). In addition, both the MSC-CM and MSC^Ang1^-CM groups exhibited an increase in skin thickness compared to PBS, but no statistical difference was found between them (Figure 3F). These data demonstrated that MSC^Ang1^-CM, compared to MSC-CM, enhanced skin healing without causing excessive skin proliferation.

### 3.4. Engineered MSC^Ang1^-CM Promotes Hair Follicle Regeneration and Angiogenesis in Diabetic Mice Wounds

**Figure 4:**
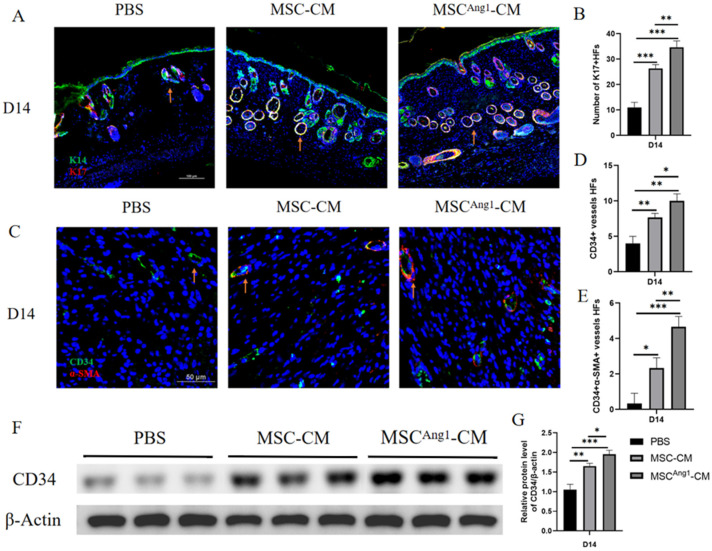
MSC^Ang1^-CM promotes regeneration of hair follicles and formation of blood vessels in diabetic mice wound migration repair area. (**A**) Dual immunofluorescence staining images of K14 and K17 in diabetic mice wounds after 14 days of different treatments. (**B**) Quantitative statistical analysis of hair follicle numbers in diabetic mice wounds on day 14 showed that compared with PBS and MSC-CM treatment, the number of K17-positive hair follicles in the migration repair area of the diabetic wound in the MSC^Ang1^-CM treatment group was significantly higher. (**C**) Dual immunofluorescence staining images of CD34 and αSMA in the wound migration repair area. (**D**,**E**) Quantitative statistical analysis of CD34+ vessels and CD34+αSMA+ double-positive mature vessel structures; (**D**) results showed that, compared to the PBS and MSC-CM groups, the MSC^Ang1^-CM treatment group had a significant increase in CD34+ vascular-like structures; (**E**) results showed that, compared to the PBS and MSC-CM groups, the MSC^Ang1^-CM treatment group was significantly increased in CD34+α-SMA+ double-positive mature vascular-like structures in diabetic mice wounds. (**F**) Western blot results showed that, after 14 days of treatment with PBS, MSC-CM, and MSC^Ang1^-CM, the MSC^Ang1^-CM group significantly upregulated the expression level of CD34 in the diabetic skin wound tissues, with β-Actin as the internal reference. (**G**) Semi-quantitative statistical analysis of CD34 showed that compared to the PBS and MSC-CM groups, the expression level of CD34 in the diabetic skin wound tissues treated with MSC^Ang1^-CM for 14 days was significantly increased, with a statistically significant difference between them. Scale bars: 50 μm, 100 μm. All data are presented as mean ± SD. * *p* < 0.05, ** *p* < 0.01, *** *p* < 0.001.

The above H&E staining revealed more hair follicles and blood vessels in the MSC^Ang1^-CM group. Therefore, double immunofluorescence staining of the epidermal basal layer marker K14 and hair follicle inner root sheath marker K17 was performed on diabetic mice wound tissues treated for 14 days with PBS, MSC-CM, and MSC^Ang1^-CM (Figure 4A). The results showed a significant increase in the number of hair follicles in the wound migration repair area following MSC^Ang1^-CM treatment (Figure 4B). CD34 is one of the markers of vascular endothelial cells, and we detected angiogenesis by immunofluorescence in situ in this study and also verified the difference in CD34 expression between different treatment groups by western blot to indirectly illustrate the angiogenesis in each group. Dual immunofluorescence staining of the endothelial cell marker CD34 and the pericyte marker αSMA indicated that MSC^Ang1^-CM treatment led to a substantial increase in CD34 and αSMA vessel-like structures (Figure 4C). Statistical analysis demonstrated that MSC^Ang1^-CM significantly promoted the generation and maturation of new blood vessels in diabetic mice wounds (Figure 4D,E). Western blot results showed the highest expression level of CD34 protein in the MSC^Ang1^-CM compared to PBS and MSC-CM, with statistical differences (Figure 4F,G). These data proved that MSC^Ang1^-CM facilitates the functional reconstruction of diabetic mice wounds by enhancing hair follicle regeneration and blood vessel formation and maturation.

### 3.5. Effect of Engineered MSC^Ang1^-CM on Fibrosis in Diabetic Wounds and Vessels

**Figure 5:**
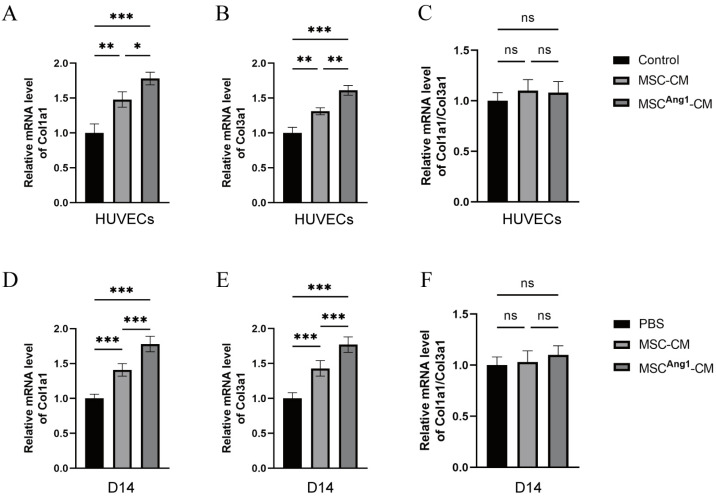
Impact of Ang1 secreted by engineered MSCs^Ang1^ on fibrosis. (**A**–**C**) Expression levels of Col1a1, Col3a1, and the Col1a1/Col3a1 ratio in HUVECs after 24 h of different treatments. The results showed that both Col1a1 and Col3a1 expression levels were elevated in the MSC^Ang1^-CM compared to Control and MSC-CM; however, there were no significant differences in the Col1a1/Col3a1 ratio. (**D**–**F**) Expression levels of Col1a1, Col3a1, and the Col1a1/Col3a1 ratio in diabetic skin wound tissues after 14 days of different treatments. The results showed that both Col1a1 and Col3a1 expression levels were elevated in the MSC^Ang1^-CM compared to PBS and MSC-CM; however, there were no significant differences in the Col1a1/Col3a1 ratio among different treatments. Control: Replacement of 50% of the original medium of HUVECs with fresh D/F medium ns *p* > 0.05, * *p* < 0.05, ** *p* < 0.01, *** *p* < 0.001.

An article suggests that excessive renin-angiotensin system activation may lead to wound fibrosis [22]. Collagen type I (COL-I) is the main structural scaffold of the skin, providing mechanical strength, while collagen type III (COL-III) gives the skin its elasticity and regulates fiber diameter and growth. The ratio of the two directly influences the regenerative and fibrotic processes of the skin: lower COL-I/COL-III ratios contribute to tissue regeneration, whereas higher ratios (5:1 in fibrosis) enhance tissue rigidity, leading to increased fiber diameter and decreased elasticity [23]. Thus, the fibrosis in diabetic mice wounds and HUVECs was evaluated. The qPCR results of collagen type 1 (Col1a1) and collagen type 3 (Col3a1) in vitro assay of HUVECs showed that both Col1a1 and Col3a1 expression levels were elevated in the MSC^Ang1^-CM compared to the Control and MSC-CM (Figure 5A,B); however, there were no significant differences in the Col1a1/Col3a1 ratio (Figure 5C). Results of Col1a1 and Col3a1 in diabetic mice wound tissues showed that both Col1a1 and Col3a1 expression levels were elevated in the MSC^Ang1^-CM compared to PBS and MSC-CM (Figure 5D,E); similarly, there were no significant differences in the Col1a1/Col3a1 ratio among different treatments (Figure 5F). These consistent in vivo and in vitro results indicated that transient Ang1 presence increases Col1a1 and Col3a1 production without causing excessive fibrosis in diabetic mice wounds and vessels.

## 4. Discussion

In this study, we prepared MSC^Ang1^-CM by genetically modifying human umbilical cord mesenchymal stem cells to overexpress Ang1 and evaluated its wound healing potential in diabetic mice. MSC^Ang1^-CM significantly enhanced wound contraction, epidermal regeneration, folliculogenesis, and angiogenesis. Compared to MSC-CM, it showed superior vascular maturation and stability, suggesting a promising cell-free diabetic wound therapy strategy.

By increasing the expression of vascular markers CD34 and α-SMA, this study validated Ang1’s critical role in diabetic wound healing. The treatment not only promoted neoangiogenesis but also improved vascular functional stability. The notable increase in hair follicle regeneration further demonstrated MSC^Ang1^-CM’s potential for tissue reconstruction.

In addition, despite the increased expression of Col1a1 and Col3a1 in the MSC^Ang1^-CM group, the ratio of Col1a1/Col3a1 did not change significantly, suggesting that the transient enhancement of MSC^Ang1^-CM promotes skin regeneration without triggering excessive fibrosis. This property has important implications for clinical applications, as the key to wound healing lies in balancing tissue regeneration and fibrosis.

Compared with existing studies, this study further expands the therapeutic potential of Ang1 [24,25,26,27]. Previous studies have shown that Ang1 plays an important role in angiogenesis through the TIE-2 receptor pathway [28,29,30], but its direct application is limited by rapid degradation and difficult dose control. In the present study, we successfully overcame these problems by genetically modifying MSCs, enabling Ang1 to be released stably and continuously in local wounds. In addition, MSC-CM has been extensively studied in promoting wound healing [31,32,33], but there is still lack of standardization in the production of secretomes, studies on modes of delivery of secretomes, and their secreted factors [17,34]. In the present study, the therapeutic effect of CM was significantly enhanced by augmenting Ang1 expression, and this optimization strategy provides new ideas to improve the existing CM treatment protocol.

Genetically modified MSCs may have complex changes in their exocytosis components, including microRNAs, metabolites, and so on. The optimization of the subsequent therapeutic effect may not be due solely to the overexpressed gene itself [35]. Therefore, we hypothesized that the promotional effect of A on skin wound healing in diabetic mice was caused by the following pathway: Ang1 promotes endothelial cell migration and lumen formation through activation of TIE-2 receptors, thereby accelerating angiogenesis [30]; the stable local release of Ang1 promotes vascular maturation and function and increases the stability of the vascular wall, contributing to long-term improvement of tissue perfusion [28]. The synergistic effect of multiple bioactive factors in MSC^Ang1^-CM, such as the co-promotion of vascular endothelial growth factor VEGF and Ang1, results in more comprehensive and effective wound healing [24]. These mechanisms validate the critical role of Ang1 in angiogenesis and further suggest that enhancing its secretion by genetic modification can significantly improve the efficacy.

Despite the positive results of this study, some limitations remain. First, the animal results of this study may face challenges in clinical translation; for example, different immune microenvironments may exist in human tissues. Second, although Ang1 overexpression showed promising short-term effects, its long-term safety and possible side effects still need to be further investigated. In addition, the preparation process of MSC^Ang1^-CM still needs to be optimized to achieve a higher stability and lower production cost. In light of the results of this study, future research could be directed towards optimizing the combination therapy of CM with other key factors, such as vascular endothelial growth factor or growth factors, to improve efficacy. Other studies could explore the potential of MSC^Ang1^-CM in other types of hard-to-heal wounds, such as radiologic injuries or burns, Or validate efficacy through large animal models to further assess the feasibility of clinical translation. In summary, the present study demonstrates that MSC^Ang1^-CM is a highly effective cell-free therapy capable of significantly promoting diabetic wound healing by enhancing angiogenesis, tissue regeneration, and maintenance of fibrotic homeostasis, demonstrating superior therapeutic potential and feasibility for clinical translation.

## 5. Conclusions

This study validated the potential of genetically modified MSC-CM in cell-free therapy by significantly improving wound healing in diabetic mice with MSC^Ang1^-CM. It provides new ideas and theoretical basis for diabetic wound treatment by promoting angiogenesis and tissue reconstruction while avoiding fibrosis.

## Data Availability

The data that support the findings of this study are available from the corresponding author upon reasonable request.

## Supplementary Materials

The following supporting information can be downloaded at: https://www.mdpi.com/article/10.3390/bioengineering11121244/s1, Table S1: Primers used for qPCR analysis.
