# Supplementary material for: The Effect of Conditioned Medium from Angiopoietin-1 Gene-Modified Mesenchymal Stem Cells on Wound Healing in a Diabetic Mouse Model
Source: Bioengineering (Basel). 2024 Dec 9;11(12):1244. doi: 10.3390/bioengineering11121244 (PMC11673525; doi:10.3390/bioengineering11121244)
Supplement: Supplementary file 1 [file bioengineering-11-01244-s001.zip › bioengineering-3268958-supplementary.pdf]

bioengineering

Article

The Effect of Conditioned Medium from Angiopoietin-1 Gene-Modified Mesenchymal Stem Cells on Wound Healing in a Diabetic Mouse Model

Qiong Deng [1,†], Shenzhen Pan [1,†], Fangzhou Du [2,3], Hongfei Sang [1], Zhixin Cai [1], Xiaoyu Xu [4], Qian Wei [2,3], Shuang Yu [2,3], Jingzhong Zhang [2,3,*] and Chenglong Li [1,*]

Table S1. Primers used for qPCR analysis.

gene name	Sequence
msGAPDH	forward, 5′-AGGTCGGTGTGAACGGATTTG-3′
	reverse, 5′-TGTAGACCATGTAGTTGAGGTCA-3′
msANG1	forward,5′-CACAGGGACAGCAGGCAAAC-3′
	reverse, 5′-AGGGCCACAGGCATCGA-3′
msCOL1A1	forward, 5′-CCTACTCAGCCGTCTGTGC-3′
	reverse, 5′-CCCTCGCTTCCGTACTCG-3′
msCOL3A1	forward, 5′-ATGCCCACAGCCTTCTACAC-3′
	reverse, 5′-GGCCAGGGTCACCATTTCTC-3′
hGAPDH	forward, 5′-AGGTCGGAGTCAACGGATTTG-3′
	reverse, 5′-TGTAAACCATGTAGTTGAGGTCA-3′
hCOL1A1	forward, 5′-CCTCAAGAGAAGGCTCACGATGGTG-3′
	reverse, 5′-AGGTCTCACCAGTCTCCATGTTGCA-3′
hCOL3A1	forward, 5′-GCCCACGTGGTGACAAAGGT-3′
	reverse, 5′-CATCTTTGCCAGGAGGTCCA-3′
